# Pervasive and non-random recombination in near full-length HIV genomes from Uganda

**DOI:** 10.1093/ve/veaa004

**Published:** 2020-02-11

**Authors:** Heather E Grant, Emma B Hodcroft, Deogratius Ssemwanga, John M Kitayimbwa, Gonzalo Yebra, Luis Roger Esquivel Gomez, Dan Frampton, Astrid Gall, Paul Kellam, Tulio de Oliveira, Nicholas Bbosa, Rebecca N Nsubuga, Freddie Kibengo, Tsz Ho Kwan, Samantha Lycett, Rowland Kao, David L Robertson, Oliver Ratmann, Christophe Fraser, Deenan Pillay, Pontiano Kaleebu, Andrew J Leigh Brown

**Affiliations:** v1 Institute of Evolutionary Biology, University of Edinburgh, Edinburgh, UK; v2 Biozentrum, University of Basel, Basel, Switzerland; v3 Swiss Institute of Bioinformatics, Basel, Switzerland; v4 Medical Research Council (MRC)/Uganda Virus Research Institute (UVRI) and London School of Hygiene and Tropical Medicine (LSHTM) Uganda Research Unit, Entebbe, Uganda; v5 Uganda Virus Research Institute, Entebbe, Uganda; v6 Department of Mathematics, Makerere University, Kampala, Uganda; v7 The Roslin Institute, University of Edinburgh, Edinburgh, UK; v8 Max Planck Institute for the Science of Human History, Jena, Germany; v9 Division of Infection and Immunity, University College London, London, UK; v10 European Molecular Biology Laboratory-European Bioinformatics Institute (EMBL-EBI), Wellcome Genome Campus, Hinxton, UK; v11 Nelson R. Mandela School of Medicine, Africa Health Research Institute, Durban, South Africa; v12 Stanley Ho Centre for Emerging Infectious Diseases, The Chinese University of Hong Kong, Shatin, Hong Kong; v13 MRC Centre for Virus Research, University of Glasgow, Glasgow, UK; v14 Department of Mathematics, Imperial College London, London, UK; v15 Nuffield Department of Medicine, Big Data Institute, Li Ka Shing Centre for Health Information and Discovery, University of Oxford, Oxford, UK

**Keywords:** HIV, genome, subtypes, phylogenetics, recombination, breakpoints

## Abstract

Recombination is an important feature of HIV evolution, occurring both within and between the major branches of diversity (subtypes). The Ugandan epidemic is primarily composed of two subtypes, A1 and D, that have been co-circulating for 50 years, frequently recombining in dually infected patients. Here, we investigate the frequency of recombinants in this population and the location of breakpoints along the genome. As part of the PANGEA-HIV consortium, 1,472 consensus genome sequences over 5 kb have been obtained from 1,857 samples collected by the MRC/UVRI & LSHTM Research unit in Uganda, 465 (31.6 per cent) of which were near full-length sequences (>8 kb). Using the subtyping tool SCUEAL, we find that of the near full-length dataset, 233 (50.1 per cent) genomes contained only one subtype, 30.8 per cent A1 (*n* = 143), 17.6 per cent D (*n* = 82), and 1.7 per cent C (*n* = 8), while 49.9 per cent (*n* = 232) contained more than one subtype (including A1/D (*n* = 164), A1/C (*n* = 13), C/D (*n* = 9); A1/C/D (*n* = 13), and 33 complex types). *K*-means clustering of the recombinant A1/D genomes revealed a section of envelope (C2gp120-TMgp41) is often inherited intact, whilst a generalized linear model was used to demonstrate significantly fewer breakpoints in the gag–pol and envelope C2-TM regions compared with accessory gene regions. Despite similar recombination patterns in many recombinants, no clearly supported circulating recombinant form (CRF) was found, there was limited evidence of the transmission of breakpoints, and the vast majority (153/164; 93 per cent) of the A1/D recombinants appear to be unique recombinant forms. Thus, recombination is pervasive with clear biases in breakpoint location, but CRFs are not a significant feature, characteristic of a complex, and diverse epidemic.

## 1. Introduction

Human immunodeficiency virus (HIV) is a highly diverse retrovirus at both the within-individual and population level (Smyth, Davenport, and Mak 2012). The HIV reverse transcriptase (RT) is error-prone resulting in a high mutation rate. RT also facilitates recombination via template switching between the two RNA genomes packaged inside the virion (Hu and Hughes 2012). The diversity of HIV allows the virus to evade host defenses, accrue drug resistance mutations, and prevent effective vaccine development (Rambaut et al. 2004).

HIV-1 Group M group contains the greatest genetic diversity. This group likely diversified in Kinshasa (Democratic Republic of Congo or DRC) from the 1920s to the 1960s, before rapidly expanding into global susceptible populations (Korber 2000; Worobey et al. 2008; Faria et al. 2014). Forming phylogenetically distinct clades, the subtypes A–D, F–H, J, and K (and sub-subtypes within e.g. A1), are found globally but frequently have broad geographic associations, mainly as the result of founder effects (Rambaut et al. 2001; Archer and Robertson 2007). Meanwhile, the DRC retained as much diversity as the global pandemic (Niama et al. 2006). As they spread, the subtypes almost certainly underwent extensive recombination throughout their evolution including at an early stage (Kalish et al. 2004; Ward et al. 2013; Olabode et al. 2019).

Recombination between different HIV variants occurs in individuals with dual infection (Robertson et al. 1995), either acquired simultaneously (co-infection) or sequentially (superinfection). This gives rise to unique recombinant forms (URFs) especially in regions where more than one subtype is common (Yebra et al. 2015; Bbosa et al. 2019). If three or more recombinant genomes without direct epidemiological linkage are found, they may be defined as a circulating recombinant form (CRF) (Robertson 2000). In addition, recombination between viruses of the same subtype (intra-subtype) occurs (Kraft et al. 2012), especially where there are high rates of dual infections (Taylor and Korber 2005), although as it is more difficult to detect due to the similarity of the recombining sequences (Yang et al. 2005) it is therefore less well documented.

HIV-1 subtypes represent major clades that have a lengthy period of distinct identity, thus assigning sequences to subtypes is inherently a phylogenetic problem. Correctly placing sequences into clades of ancestral diversity relies on the availability of representative reference sequences, that themselves are unrecombined and correctly classified. It is made challenging by growing global diversity, the accumulation of drug resistance mutations (essentially equating to convergent evolution), and in particular, widespread recombination. Manual phylogenetics has been described as a ‘gold standard’ for subtype classification (Pineda-Peña et al. 2013; Fabeni et al. 2017), but a number of automated tools exist (de Oliveira et al. 2005; Struck et al. 2014) which are particularly useful in subtyping large datasets and databases.

Automated subtyping methods have good accuracy compared to manual phylogenetics in the case of the simple ‘pure’ subtype using just the *pol* region (Pineda-Peña et al. 2013; Fabeni et al. 2017), although a similar assessment has not been undertaken for whole-genome tools. Agreement between methods is better for certain subtypes (e.g. B or C), whilst more challenging for others (e.g. A or D), and novel recombinants with sections of different phylogenetic history are a particular source of disagreement (Gifford et al. 2006), highlighting the inherent difficulties in classifying recombinant sequences. The description of new CRFs for instance, typically involves showing that sequences form a monophyletic cluster amongst a background of other sequences, followed by a ‘boot-scanning’ sliding window approach (Salminen et al. 1995) to find putative sections of different subtypes, and then a more detailed and laborious confirmation by hand: for example (Carr et al. 1998; Foster et al. 2014).

SCUEAL (Kosakovsky Pond et al. 2009) is an automated tool, which finds the most likely subtype or recombinant mosaic with a model-based evaluation. Briefly, a reference set of pure subtypes and CRF genomes is used to make a reference alignment, tree, and an inferred root sequence which remains constant for each query and model proposal. The query sequence is then aligned to the inferred root sequence, grafted to the reference set to make a three-taxon tree, and the maximum-likelihood placement is found. A genetic algorithm acts upon a population of models to create mosaic suggestions for a fixed number of breakpoints. BIC is used to assess the fitness of models in the population, which evolve until there is no improvement after several generations (the stopping criteria). Additional breakpoints may be added until there is no further BIC improvement (and a step-down verification). Model averaged support for the best mosaic is found using the sum of Akaike weights of all concordant proposed models. A 95% confidence interval for the breakpoint location is found using a similar principle.

In Uganda, HIV was prevalent by the early 1980s (Serwadda et al. 1985). Two circulating subtypes (A1 and D) are present at similar frequencies in the population (Yirrell et al. 1998, 2002), alongside unique A1/D recombinants (Eshleman et al. 2002). These two subtypes are thought to represent independent introductions of HIV diversity into Uganda, with A1 having arrived first via the rural south-west in the 1950s or 60s, followed later by subtype D about 10 years later (Yebra et al. 2015). There were already reports of growing numbers of AIDS cases (then identified as aggressive Kaposi’s sarcoma or slim disease) in the rural Rakai region of south western Uganda in the 1970s (Serwadda et al. 1986; Kuhanen 2010). Surveillance studies found seropositivity in 1987 in pregnant women attending hospitals in the capital, Kampala, was 24.1 per cent (Carswell 1987). Today the adult prevalence is estimated to be within 5.7 and 6.2 per cent (Joint United Nations Programme on HIV/AIDS 2019; Ministry of Health Uganda 2019). Dual infections can be found in female sex workers (Ssemwanga et al. 2012; Redd et al. 2014) and also at substantial levels in general population and low risk rural cohorts (Kiwanuka et al. 2010; Ssemwanga et al. 2011; Redd et al. 2012). Therefore, subtypes A1 and D have been co-circulating in Uganda for perhaps as long as 50 years, with high rates of incidence and dual infection, providing ample opportunity for recombination to occur.

The PANGEA-HIV project (Pillay et al. 2015) was set up with the aims of using phylogenetics to better understand the dynamics and drivers of ongoing transmission in African HIV epidemics and has generated large numbers of near full-length genome sequences. The data generated with samples obtained by MRC/UVRI in Uganda presented an opportunity to study the prevalence of recombinants and the distribution of their breakpoint locations along the genome in a population setting, using numerical breakpoint locations from SCUEAL models.

## 2. Methods

### 2.1 Sample collection

Samples were collected by the MRC/UVRI and LSHTM Uganda Research Unit between 2007 and 2017 from sites and cohorts across southern Uganda. These included the Masaka District in the rural South West, female sex workers from Kampala, and people living in fishing communities on the shores and islands around Lake Victoria. Ethical approval was given by the Uganda Virus Research Institute Research and Ethics Committee (UVRI-REC, Federal Wide Assurance (FWA) No. 00001354), the Uganda National Council for Science and Technology (UNCST FWA No. 00001293), and the University of Edinburgh School of Biological Sciences Ethics Committee (12 June 2018). All participants were recruited voluntarily and provided written informed consent.

### 2.2 Sequencing and alignment

Viral RNA was extracted from plasma by automated extraction. Near full-length HIV-1 genomes were reverse transcribed and amplified in four overlapping amplicons using a one-step RT-PCR protocol and a pan-HIV-1 primer set (Gall et al. 2012). Amplicons were pooled in equimolar amounts and sequenced using Illumina MiSeq 250-bp paired-end technology as previously described in Gall et al*.* (2014).

Consensus sequences were generated from short reads using an in-house *de novo* assembly pipeline as follows. Trimmomatic (Bolger, Lohse, and Usadel 2014) was used to trim reads using a mean Phred quality score cut-off of 30. Human reads were removed by mapping to a smalt [www.sanger.ac.uk/science/tools/smalt-0; last accessed 7 January 2020], index consisting of HIV genomes [downloaded from GenBank: www.ncbi.nlm.nih.gov/genbank; last accessed 7 January 2020], and the hg38 human assembly [downloaded from Ensembl: ensembl.org; last accessed 7 January 2020]: read pairs where either or both reads mapped to hg38 were removed. *De novo* assembly was then performed using Iterative Virus Assembler (Hunt et al. 2015), and contigs aligned to their closest viral reference using lastz (Harris 2007). Custom Perl scripts were then used to concatenate contigs into draft genomes and subsequently generate consensus sequences by a process of iterative mapping using smalt and SAMtools (Li et al. 2009). We applied a read depth cut‐off of ≥20 reads to these final genomic sequences before subsequent analyses.

In total 1,277 consensus genome sequences were produced at the Wellcome Sanger Institute, following the above protocol. In addition, 603 consensus genomes were produced using a similar approach by the Africa Centre (Durban, South Africa). After removal of duplicates the dataset comprised 1,857 sequences. Of these, 1,472 (79.3 per cent) were over 5,000 bp, 1,218 (65.6 per cent) were over 6,000 bp, 797 (42.9 per cent) were over 7,000 bp, and 465 (25.0 per cent) were near full length at over 8,000 bp which were used in the breakpoint analyses. Of these last, 371 were sequenced at the Wellcome Sanger Institute and 94 sequenced at the Africa Centre. The consensus sequences were aligned using MAFFT (Katoh and Standley 2013), and where necessary manually edited after visual inspection. The alignment starts from the first codon of *gag* (HXB2, 790) and ends at the last codon of *nef* (HXB2, 9,415). Hypervariable loops 1 + 2, 4, and 5 in *env* (HXB2 6,615–6,812; 7,377–7,478; 7,599–7,637) were removed from the alignment as these can rarely be aligned with confidence (Simmonds et al. 1990). The sequences are submitted to Genbank under the accession numbers MN788736: MN790202.

### 2.3 Subtyping

Preliminary subtyping investigations were carried out with COMET (Struck et al. 2014), REGA (de Oliveira et al. 2005), and SCUEAL (Kosakovsky Pond et al. 2009). To compare the three, which have very different outputs, the results had to simplified. Our comparison of these three methods (Supplementary Table S1) found overall agreement to be 36 per cent (40 per cent of sequences agreed between two methods, and 24 per cent had no agreement). Where there was agreement between the three methods, these sequences tended to be pure subtypes (81 per cent), while disagreements were more common for recombinants, and any sequences with large gaps. Arau, Martins, and Oso (2019) carried out a similar comparison, but used different simplification rules, and therefore found different degrees of agreement. Of these methods however, only SCUEAL outputs breakpoint location numerically. For that reason, subtyping and breakpoint detection were undertaken with SCUEAL implemented locally using 218 full-length subtypes and CRFs as references (accession numbers in Supplementary Table S2), allowing the programme to find recombinant fragments of 300 bp and above, with a maximum number of ten breakpoints. The genetic algorithm population size was set to 128 models and was said to have converged after no score improvements in fifty generations. A validation exercise was undertaken by creating ten random A1/D *in* *silico* recombinants and analysing them one hundred times in SCUEAL to test its reliability and accuracy (Supplementary Fig. S1). The raw SCUEAL output was edited in R (R Core Team 2019) using the packages *ape* v.5.3 (Paradis and Schliep 2019), and *seqinr* v.3.6-1 (Charif and Lobry 2007) to make the following adjustments. First, SCUEAL reports breakpoints at the location in the individual sequence, not the alignment, so these were adjusted to correspond to alignment positions. Second, phylogenetic subtyping methods sometimes have difficulty distinguishing subtypes B and D in recombinants, owing to their closer common ancestry than other subtypes (Korber 2000). As no pure subtype B sequences have been observed from Uganda (Lihana et al. 2012) and subtype B was only ever seen as fragments in complex recombinants, B calls were changed to D. Similarly, we did not attempt to distinguish A2 fragments from A1, as while A1 has been established in Uganda for decades, other A lineages have not been described. Confidence intervals of individual breakpoints have been stripped for clarity. Intra-subtype breakpoints were also removed.

### 2.4 Identification of transmitted breakpoints

A maximum-likelihood tree of all A1/D recombinant genomes, three A1 sequences, and three D sequences was constructed using IQ-TREE (Nguyen et al. 2015) with fast model selection (Kalyaanamoorthy et al. 2017), to identify any obvious CRFs. The SCUEAL assessment was plotted alongside the phylogenetic tree using R packages *ape* v.5.3 (Paradis and Schliep 2019) and *phytools* v.0.6-99 (Revell 2012). Similarly, a second tree was also constructed including the non-A1/D recombinants.

To distinguish between transmitted breakpoints and independent recombination events, we used a window-based approach to find pairs of sections of the genome linked by a low genetic distance. If a given pair of genomes contained multiple consecutive linked windows and a similar breakpoint was also found inside one of these windows, it was taken as evidence for a transmitted breakpoint.

Custom R scripts were used to split genomes into 27 non-overlapping 300 bp windows and to find linkage with a threshold of 2 per cent divergence using the TN93 nucleotide distance (Tamura and Nei 1993). This is similar to the HIV-TRACE approach (Kosakovsky Pond et al. 2018), but considers multiple windows instead of the whole sequence. This approach was tested with randomly generated recombinants (see Supplementary Fig. S2), and it was shown that at the 2 per cent level, some references would be linked in some single windows. This 2 per cent threshold was slightly higher than the usual 1.5 per cent threshold often used in studies of transmission clusters using *pol* sequences for example (Mehta et al. 2015). There is no set distance that a pair of CRF genomes might be linked to each other: it will depend on the time since recombination and subsequent spread (younger CRFs should have lower thresholds). The purpose of this linkage was not to find recent transmission pairs, but to find sections of the genome that were related and shared a clearly transmitted breakpoint. All of the A1/D recombinant pairs linked by more than two out of twenty-seven windows at the 2 per cent level were examined. Where there was evidence for transmitted breakpoints between pairs of genomes, only one genome was kept in the subsequent generalized linear model (GLM) analysis to avoid issues of non-independence.

### 2.5 Recombination pattern classification

To classify A1/D recombinant genomes, each genome was transformed into binary characters identifying subtype at each nucleotide position (A1 recorded as 0, D recorded as 1). A Euclidean distance matrix was generated from the recoded data and *K*-means clusters were found using the *kmeans* function from the package *stats* v.3.6.0 (part of base R) and the algorithm of Hartigan and Wong (1979), which divides the data into groups by minimizing within-cluster variation. The optimal value of *K* was judged with the gap statistic (Tibshirani, Walther, and Hastie 2001), and the elbow and silhouette methods using the *cluster* v.2.0.8 (Maechler et al. 2019) and *factoextra* v.1.0.5 (Kassambara and Mundt 2017) R packages.

### 2.6 Breakpoint and genome location model framework

Breakpoints of all inter-subtype recombinant genomes at different genome positions were analysed using a generalized linear model in R. The binary response was presence or absence of a breakpoint, aggregated for each window of the genome, transformed with the logit link. Genomes were divided into twenty-seven windows of 300 bp in length. The first window did not contain breakpoints (as the minimum length to assign a subtype was constrained to 300 bp), and the last window was fewer than 300 bp. Both were removed from the analysis. Following the genome *K-*means clustering result, the genome regions were defined into three broad regions of the genome. These were 1, windows containing gag–pol (windows 1–13), 2, a custom region of envelope (C2-TM, from C2 of gp120 to the transmembrane region of gp41, windows 19–22), and 3, accessory gene regions (*vif, vpr, vpu,* 14–18) and the cytoplasmic tail of gp41 plus *nef* 22–26.

## 3. Results

### 3.1 Subtype distribution

**Figure 1.**
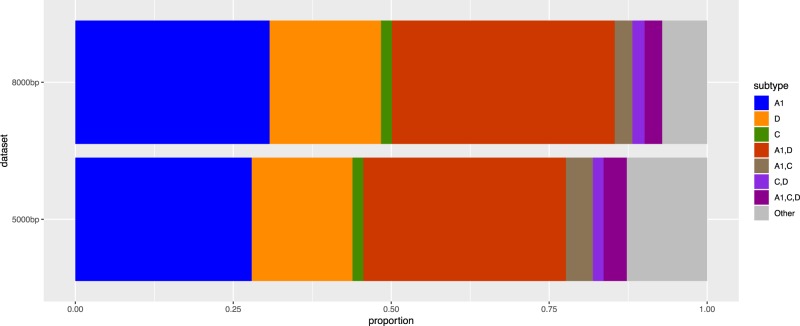
Subtype distribution in the 5,000 bp and above genomes, *n* = 1,472, and the near full length 8,000 bp and above dataset, *n* = 465.

The MRC PANGEA-HIV genome dataset comprised 1,857 sequences, of which 1,472 were over 5,000 bp and 465 were over 8,000 bp. The subtype distribution for the 5,000 bp dataset was: 411 (27.9 per cent) A1, 235 (16.0 per cent) D, 25 (1.7 per cent) C, 472 (32.1 per cent) A1/D, 63 (4.3 per cent) A1/C, 25 (1.7 per cent) C/D, 54 (3.7 per cent) A1/C/D, and 187 (12.7 per cent) complex. Of the 465 near full-length genomes, 233 (50.1 per cent) were ‘pure’ containing only one subtype (143 A1; 82 D; 8 C), while 232 (49.9 per cent) were inter-subtype recombinants (164 A1/D; 13 A1/C; 9 C/D; 13 A1/C/D; and 33 other complex recombinants Fig. 1). SCUEAL called more ‘complex’ and ‘other’ subtypes in the 5,000 bp dataset than the more complete sequences, which may be due to gaps in the sequence. Excluding the ‘complex’ category however, there was no difference in subtype proportions between these two datasets (χ^2^ = 4.19, df = 6, P = 0.65), and the ratio of A1 to D genomes was similar (1.743:1 in the 8,000 bp and 1.748:1 in the 5,000 bp dataset), confirming a lack of bias in successful sequencing by subtype or recombinant status. For the remaining analyses, we used the near full-length genome dataset where subtype and location of breakpoints could be most accurately determined.

### 3.2 Identification of CRFs and transmitted breakpoints

**Figure 2.**
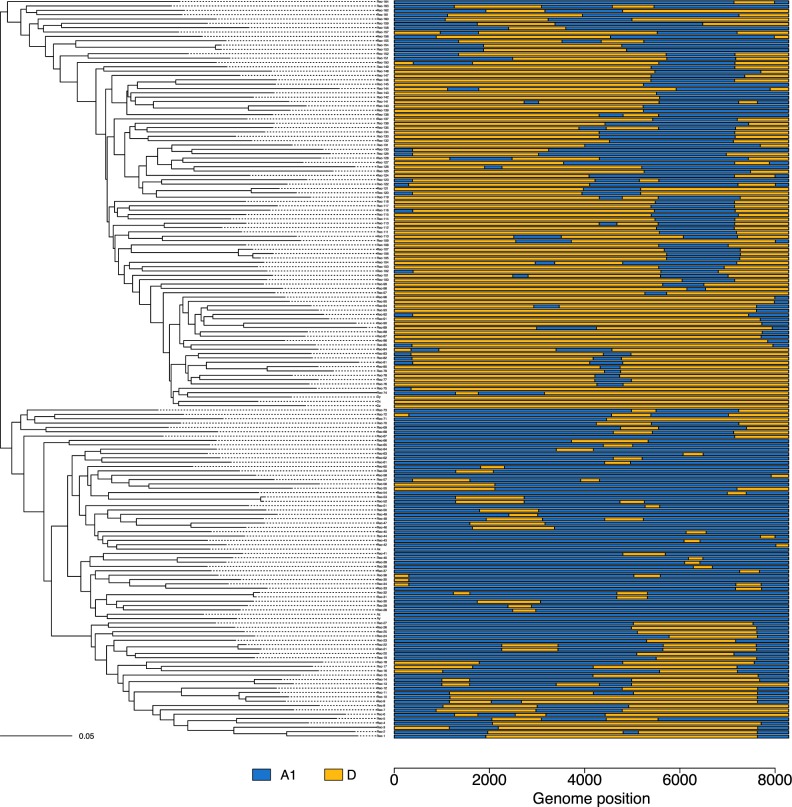
Maximum-likelihood reconstruction of the A1/D recombinants using IQ-TREE and their SCUEAL subtype (right). One triplet (Rec-105 to Rec-107), and a few cherries can be seen (e.g. Rec-153 and Rec-154). Some examples of convergent recombination patterns include Rec-116 and Rec-147, Rec-8 and Rec-160, Rec-29 and Rec-158.

A maximum-likelihood tree of the A1/D recombinants with three A1 and D pure sequences was constructed (Fig. 2). A similar figure is presented for non-A1/D recombinants (*n* = 68) in Supplementary Fig. S3. Although the overall phylogeny is confounded by the violation of the key assumption that there are no recombinants, any CRF should form a clear monophyletic cluster.

Midpoint rooting broadly splits the tree into genomes predominantly containing subtype D, and those predominantly containing subtype A1 (the three references of each subtype fall within these respective groups). There are a few closely related cherries, and one closely related triplet (Fig. 2). Notably, some recombinants with a similar recombinant pattern can be found on altogether different parts of the tree, showing clear evidence of convergent recombination (e.g. Rec-116 and Rec-147, Rec-8 and Rec-160, Rec-29 and Rec-158).

**Figure 3.**
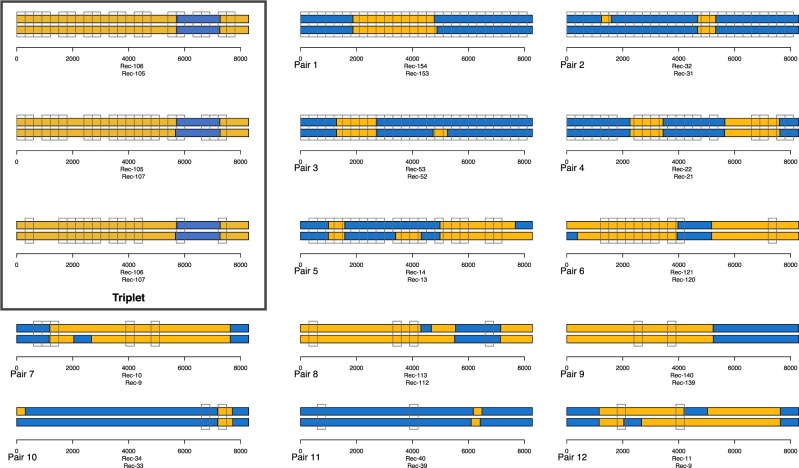
Pairs of genomes linked by a distance of less than 2 per cent genetic distance (TN93) in two or more 300 bp windows along the genome. The matching windows are shown with open clear boxes, and the SCUEAL subtyping result for the genome pairs are in colour (blue for subtype A1 and orange for subtype D).

We then used a window-based approach to find consecutive genetically linked windows that contained similar breakpoints, in an attempt to distinguish transmitted and unique breakpoints. Of the 164 A1/D recombinants, there were twelve single pairs, linked at a 2 per cent threshold in a minimum of two out of twenty-seven windows (Fig. 3). There were also pairs forming a triplet (boxed), which had a similar recombination pattern in all three sequences and was tightly linked in multiple windows. However, there is epidemiological linkage of two of these sequences (data not shown) and therefore it does not meet the requirements of a CRF.

Pairs 1–3 were linked in twenty-seven/twenty-seven windows and are likely to have been transmitted relatively recently. Pair 2 has an almost identical subtype result and those breakpoints were probably transmitted. Other matching breakpoints outside of linked windows (e.g. in pair 4 or 6) could represent transmitted breakpoints whose windows have diverged sufficiently to indicate an older common ancestor.

Assuming there is evidence for transmitted breakpoints in pairs 1–12 (the A1/D pairs) and the triplet, there are fourteen A1/D genomes that have evidence for being transmitted wholly or partially, and these pairs and triplet can be found as closely linked tips in the phylogenetic tree (Fig. 2). Overall, as the vast majority of the A1/D genomes (150/164; 91 per cent) lack linkage with other genomes, we see no evidence for large-scale transmission of individual recombinants such as would be recognized as a CRF, and so all should be considered URFs. Linked windows with non-matching breakpoints (e.g. pairs 1, 3, 5) are likely to represent competing SCUEAL models with similar likelihoods, perhaps in regions where divergent subtypes are more similar.

### 3.3 Recombinant groupings

**Figure 4.**
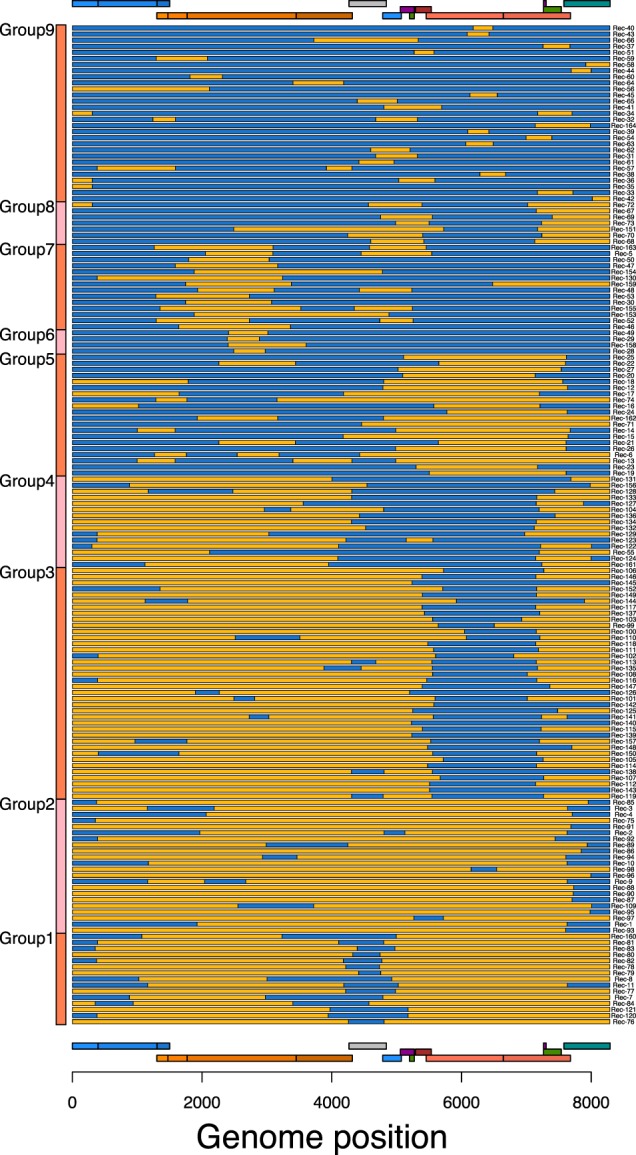
Recombination pattern of the A1/D recombinant genomes (*n* = 164). Genome position is on the *x*-axis and each horizontal bar is an individual genome recombination pattern. Segments of orange colour represent subtype D, while blue colouration represents subtype A1.

The A1/D recombinants were placed into groups to highlight similarities in recombination patterns. This was done by putting subtype identity at each position along each genome through a *K*-means clustering algorithm. The optimum number of groups was found to be nine. Figure 4 shows a representation of the 164 A1/D recombinant genomes placed into these nine groups (see Supplementary Figs S4–S6 for justification of, and alternative values of *K*). Group 1 contains mostly subtype D (in orange) with small sections of subtype A1 (in blue), whereas group 9 contains mostly subtype A1 with small sections of subtype D. In the remaining groups it is notable that a section of envelope appears to be inherited intact in many A1/D recombinants. This was observed in both directions, where subtype A1 envelope was found on a background of subtype D (groups 3–5), and subtype D envelope was found on a background of subtype A1 (groups 6 and 7). The part of envelope these groups have in common spans from the C2 part of gp120 through to the transmembrane domain of gp41 (abbreviated C2-TM). In groups 7 and 8 the intact region of envelope extended into *nef* and there also appeared to be sections of subtype D RT (within *pol*) with A1 subtype either side.

### 3.4 Breakpoint distribution

**Figure 5.**
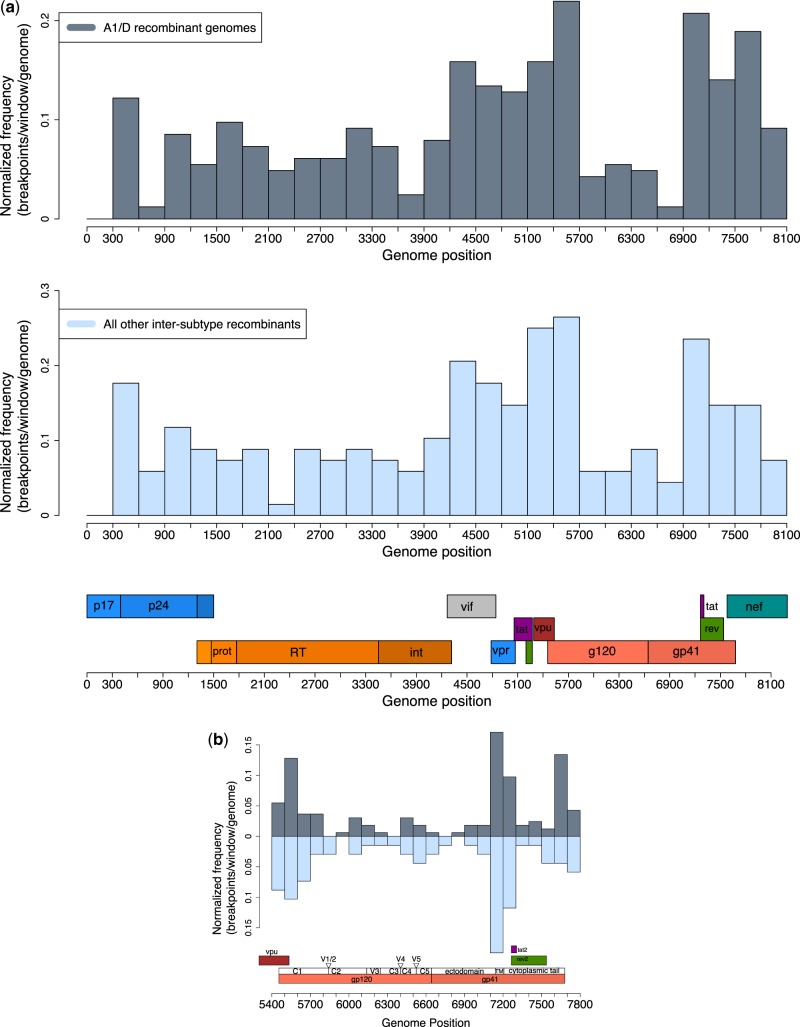
(a) Distribution of inter-subtype recombination breakpoints divided into 300 bp bins in A1/D recombinants (*n* = 164) and all other inter-subtype recombinant genomes (*n* = 68). Genome position numbering corresponds to the alignment as described in Section 2. (b) Distribution of breakpoints in the envelope region. Breakpoints have been binned into 100 bp regions and the finer sub-structure of gp120 and gp41 is shown.

The distribution of breakpoints along the genome for the A1/D genomes (*n* = 164) and all other inter-subtype recombinants genomes (*n* = 68) is shown in 300 bp windows in Fig. 5a. The two distributions were strongly positively correlated (Pearson correlation, *R*^2^ = 0.91, df =25, P < 0.001).

Both distributions show a relatively large frequency of breakpoints in the accessory gene region (covering *vif*, *vpu*, *vpr*, tat1, rev1, and genome positions 4,200–5,700), lower levels of recombination in the gag–pol region, and a particularly low level of recombination in the envelope region which was also seen in the *K*-means clustering result (Fig. 4). Figure 5b shows the distribution within envelope at a finer scale (100 bp windows) and a lower frequency of recombination within the C2-TM region (windows 20–23).

Table 1 shows the GLM summary. Regions of the genome containing gag–pol had significantly (P < 0.001) fewer breakpoints per 300 bp window per genome than the accessory gene region, as did the C2-TM region (P < 0.001). On the data scale the model finds the following estimates of breakpoint per 300 bp window per genome: gag–pol 0.073 (95% CI 0.064–0.083), env-C2-TM 0.046 (95% CI 0.035–0.062), and the accessory regions 0.166 (95% CI 0.150–0.182).

**Table 1.** Beta estimates for the GLM on the log-odds scale.

	Estimate	SE	z	P
Intercept (gene region = accessory)	−1.61635	0.05886	−27.462	<0.001
Gene region = gag–pol	−0.92597	0.09147	−10.123	<0.001
Gene region = env C2-TM	−1.40804	0.16688	−8.437	<0.001

## 4. Discussion

Multiple studies using single gene regions for example (Yirrell et al. 1998, 2002; Kaleebu et al. 2000) have previously described the HIV diversity in Uganda as predominantly subtypes A1, D, and A1/D recombinants (including A1/D URFs (Eshleman et al. 2002)). A more recent study suggests that in the *pol* region, around 15 per cent of sequences are detectable inter-subtype recombinants (Bbosa et al. 2019), however, near full-length genomes reveal substantial additional recombination: we observe here that around half (49.9 per cent) of the genomes are inter-subtype recombinants, and that most of these are URFs. Earlier small-scale studies of full-length genomes from Uganda have also shown high numbers of inter-subtype recombinants for example (14/46; 30 per cent) (Harris et al. 2002) and (92/200; 46 per cent) (Lee et al. 2017), also predominantly containing A1 and D subtypes.

This dataset, containing large numbers of near full-length sequences from a country already known to contain high numbers of unique recombinants, provided a difficult subtyping challenge. SCUEAL is an automated tool, unique in its ability to find a model-based assessment of recombination, including breakpoint locations. We have tested SCUEAL against *in silico* PANGEA subtypes A1 and D recombinant sequences, and found it to perform extremely well. Further to this, extensive tests were included in the original SCUEAL publication (Kosakovsky Pond et al. 2009), including a test against simulated recombinants, of database sequences, and in a comparison with the boot-scanning tool REGA. While it was shown to perform very well under a wide range of scenarios, accuracy wanes under the most complex scenarios, for instance those with more breakpoints, with closely related recombining sequences, and short fragments. Whilst SCUEAL is an extremely powerful model-based estimation of recombinant history, it is probabilistic, and should be interpreted as such.

According to the SCUEAL models of this dataset, there are significantly lower levels of recombination breakpoints in the *gag–pol* and envelope C2-TM regions compared with the accessory gene regions of the genome. The pattern of breakpoint frequency along the genome is remarkably similar to those in CRFs and URFs from publicly available datasets (Fan, Negroni, and Robertson 2007). These authors were the first to hypothesize that envelope is often inherited intact, being transferred into new genomes as an integral unit (Archer et al. 2008). Functional constraints of protein and RNA folding could drive these patterns, as has been shown *in vitro* (Galli et al. 2010), and this appears particularly pertinent in the envelope region, where the *K*-means clustering and GLM result showed that the C2-TM region is often inherited intact. The gp120 protein is essential for cellular entry and for outcompeting other strains (Marozsan et al. 2005), and its recombination is likely to come up against functional constraints (Simon-Loriere et al. 2009). The three-dimensional structure of envelope shows the interdependence of the gp120 and gp41 proteins, and the disruption of internal residue contacts is expected to decrease the fitness of recombinants (Woo, Robertson, and Lovell 2014). The intricate interdependences of *env* proteins have been further demonstrated *in vitro* (Bagaya et al. 2015), and also by computational simulations of protein folding (Golden et al. 2014).

Sequence identity (Baird et al. 2006; Archer et al. 2008) and RNA structure (Galetto et al. 2004) have been shown to predict recombination frequency along the HIV genome. RNA structures have also been shown to potentially enable the recombination of envelope (Simon-Loriere et al. 2010), and in particular, a hairpin in C2 is identified as a driver of recombination. This mechanistic explanation of recombination in envelope, taken together with the seemingly universal breakpoint pattern and in the global CRF datasets, may suggest the genome recombination pattern and the recombination of C2-TM as an integral unit as observed here, is not unique to Uganda, but may be generalized to other population settings.

Finding potential CRFs among a myriad of recombinant genomes is not straightforward as standard phylogenies are violated by recombination, but sequences that have a more recent common ancestor (such as CRFs) should be identifiable as a cluster. However, independent recombination events with convergent recombination patterns involving the same subtypes and breakpoints will be difficult to distinguish from CRFs that originated years or decades ago. It is also possible that some recombination events are sequential, where recombinant genomes undergo new recombination, creating breakpoints of different ages in the same genome.

We searched all recombinant sequences for shared breakpoints which would suggest recombinants had been transmitted. The error associated with breakpoint assignment in SCUEAL will be related to diversity in the surrounding region. Any case where transmission of a recombinant had occurred would lead to the flanking sequences either side of the breakpoint being homologous even if subsequent recombination caused the descendent sequences to be relocated in the phylogeny. Given the difficulty of applying phylogenetic approaches we estimated simple genetic identity across the breakpoint between putative examples of transmitted recombinants. This revealed a small number which could be assigned to linked pairs. Overall 91 per cent of these recombinants are unique, as previously seen in *pol* sequences (Yebra et al. 2015), and parallels the general low frequency of transmission pairs in the Ugandan general population (Bbosa et al. 2019). A high prevalence of URFs in Uganda and neighbouring Kenya has been seen in earlier studies (Harris et al. 2002; Yang et al. 2004; Lee et al. 2017) pointing to their continual creation, which would require a relatively high dual infection rate. In general, this would be expected to be found in transmission networks of higher degree than observed here (we found only twelve linked pairs and a triplet in a pool of 164 A1/D recombinants). It appears from this inconsistency that the HIV transmission network structure in Uganda is more complex than generally thought.

This study collapses quasi-species diversity into single consensus genomes, which may obscure recombinational variants. This would be particularly true in recent superinfections where it might be possible to find the parental strains alongside a multitude of recombinants. Song et al. (2018) skilfully made use of single-genome sequencing to explore recombination within an infection. Applied in this context it might allow us to distinguish older transmitted recombinants from those *de* *novo* within-patient.

The distinct lack of CRFs in the dataset suggests recombinants are unable to establish in any appreciable way. A recombinant might be transmitted widely if it has some biological advantage (Turk and Carobene 2015) or after going through a bottleneck in a new susceptible population for example CRF01_AE (Li et al. 2017), but neither appears to hold true in this already established and diverse epidemic. However, since the sampling density is low and only a small sample of closely linked pairs of genomes was found, our findings could also be consistent with the presence of circulating recombinants at low frequency.

Recombination is an important evolutionary force, observable at every scale, from within-patient (Song et al. 2018) to deep in HIV evolutionary history, before even the divergence of the subtypes (Olabode et al. 2019). Significant efforts have been made to quantify the general population level of recombination in HIV-1 using coalescent-based estimators (McVean, Awadalla, and Fearnhead 2002; Taylor and Korber 2005) which concluded that it can be extremely high, particularly in comparison with other viruses with comparable levels of population nucleotide diversity (e.g. HCV). Taylor and Korber extended their analysis to estimate possible levels of superinfection consistent with both the within-individual recombination level they inferred and that of the frequency of recombination inferred at the population level. They suggested that the superinfection level could be as high as 15 per cent in some combinations of parameter values. However, as they pointed out, they did not consider non-random mixing in the population, which generally applies to sexual networks (Liljeros et al. 2001).

Here, we have shown pervasive levels of inter-subtype recombination in Uganda. While at the population level some patterns of recombination breakpoints are more prevalent than expected, the effect is not large, and certainly has not given rise to outgrowth of any particular recombinant, or CRF, as the great majority are unique. A major assumption of any phylogenetic analysis is that no recombination between sequences has taken place. The greatest impact of the inferred high level of recombination in the dataset therefore appears to lie on the reconstruction and interpretation of HIV phylogenies. This may be especially true for sequences with overlooked intra-subtype recombination.

## Data availability

Sequence data analysed in this work have been submitted to GenBank under accession numbers MN788736: MN790202. The whole-genome version of SCUEAL is available on Github (https://github.com/veg/hyphy-analyses).

## Supplementary Material

veaa004_Supplementary_Data

## References

[veaa004-B1] Arau P. M. M., Martins J. S., Oso N. S. (2019) ‘SNAPPy: A Snakemake Pipeline for Scalable HIV-1 Subtype by Phylogenetic Pairing’, Virus Evolution, 5: 1–8. 10.1093/ve/vez050 PMC6863187 31768265

[veaa004-B2] Archer J., Robertson D. L. (2007) ‘Understanding the Diversification of HIV-1 Groups M and O’, AIDS, 21: 1693–700. 17690566 10.1097/QAD.0b013e32825eabd0

[veaa004-B3] Archer J. et al (2008) ‘Identifying the Important HIV-1 Recombination Breakpoints’, PLoS Computational Biology, 4: e1000178. 18787691 10.1371/journal.pcbi.1000178 PMC2522274

[veaa004-B4] Bagaya B. S. et al (2015) ‘Functional Bottlenecks for Generation of HIV-1 Intersubtype Env Recombinants’, Retrovirology, 12: 1–17. 25997955 10.1186/s12977-015-0170-8 PMC4445978

[veaa004-B5] Baird H. A. et al (2006) ‘Influence of Sequence Identity and Unique Breakpoints on the Frequency of Intersubtype HIV-1 Recombination’, Retrovirology, 3: 91. 17164002 10.1186/1742-4690-3-91 PMC1764423

[veaa004-B6] Bbosa N. et al (2019) ‘Phylogeography of HIV-1 Suggests That Ugandan Fishing Communities Are a Sink for, Not a Source of, Virus From General Populations’, Scientific Reports, 9: 1–8. 30705307 10.1038/s41598-018-37458-x PMC6355892

[veaa004-B7] Bolger A. M., Lohse M., Usadel B. (2014) ‘Trimmomatic: A Flexible Trimmer for Illumina Sequence Data’, Bioinformatics, 30: 2114–20. 24695404 10.1093/bioinformatics/btu170 PMC4103590

[veaa004-B8] Carr J. K. et al (1998) ‘Full Genome Sequences of Human Immunodeficiency Virus Type 1 Subtypes G and A/G Intersubtype Recombinants’, Virology, 247: 22–31. 9683568 10.1006/viro.1998.9211

[veaa004-B9] Carswell J. W. (1987) ‘HIV Infection in Healthy Persons in Uganda’, AIDS (London), 1: 223–7. 3126769

[veaa004-B10] Charif D., Lobry J. R. (2007) ‘SeqinR 1.0-2: A Contributed Package to the R Project for Statistical Computing Devoted to Biological Sequences Retrieval and Analysis’, in Bastolla U. et al (eds) Structural Approaches to Sequence Evolution: Molecules, Networks, Populations, pp. 207–32. Berlin, Heidelberg: Springer Berlin Heidelberg.

[veaa004-B11] de Oliveira T. et al (2005) ‘An Automated Genotyping System for Analysis of HIV-1 and Other Microbial Sequences’, Bioinformatics, 21: 3797–800. 16076886 10.1093/bioinformatics/bti607

[veaa004-B12] Eshleman S. H. et al (2002) ‘Identification of Ugandan HIV Type 1 Variants With Unique Patterns of Recombination in Pol Involving Subtypes A and D’, AIDS Research and Human Retroviruses, 18: 507–11. 12015904 10.1089/088922202317406655 PMC2573392

[veaa004-B13] Fabeni L. et al (2017) ‘Comparative Evaluation of Subtyping Tools for Surveillance of Newly Emerging HIV-1 Strains’, 55: 2827–37. 10.1128/JCM.00656-17 PMC5648718 28701420

[veaa004-B14] Fan J., Negroni M., Robertson D. L. (2007) ‘The Distribution of HIV-1 Recombination Breakpoints’, Infection, Genetics and Evolution, 7: 717–23. 10.1016/j.meegid.2007.07.012 17851137

[veaa004-B15] Faria N. R. et al (2014) ‘The Early Spread and Epidemic Ignition of HIV-1 in Human Populations’, Science, 346: 56–61. 25278604 10.1126/science.1256739 PMC4254776

[veaa004-B16] Foster G. M. et al (2014) ‘Novel HIV-1 Recombinants Spreading Across Multiple Risk Groups in the United Kingdom: The Identification and Phylogeography of Circulating Recombinant Form (CRF) 50-A1D’, PLoS One, 9: e83337–10. 24454702 10.1371/journal.pone.0083337 PMC3893077

[veaa004-B17] Galetto R. et al (2004) ‘The Structure of HIV-1 Genomic RNA in the gp120 Gene Determines a Recombination Hot Spot *In Vivo*’, Journal of Biological Chemistry, 279: 36625–32. 15218022 10.1074/jbc.M405476200

[veaa004-B18] Gall A. et al (2012) ‘Universal Amplification, Next-Generation Sequencing, and Assembly of HIV-1 Genomes’, Journal of Clinical Microbiology, 50: 3838–44. 22993180 10.1128/JCM.01516-12 PMC3502977

[veaa004-B19] Gall A. et al (2014) ‘Complete Genome Sequence of the WHO International Standard for HIV-1 RNA Determined by Deep Sequencing’, Genome Announcements, 2: 10–1. 10.1128/genomeA.01254-13 PMC3916492 24503998

[veaa004-B20] Galli A. et al (2010) ‘Patterns of Human Immunodeficiency Virus Type 1 Recombination *Ex Vivo* Provide Evidence for Coadaptation of Distant Sites, Resulting in Purifying Selection for Intersubtype Recombinants During Replication’, Journal of Virology, 84: 7651–61. 20504919 10.1128/JVI.00276-10 PMC2897624

[veaa004-B21] Gifford R. et al (2006) ‘Assessment of Automated Genotyping Protocols as Tools for Surveillance of HIV-1 Genetic Diversity’, AIDS, 20: 1521–9. 16847407 10.1097/01.aids.0000237368.64488.ae

[veaa004-B22] Golden M. et al (2014) ‘Patterns of Recombination in HIV-1M Are Influenced by Selection Disfavouring the Survival of Recombinants with Disrupted Genomic RNA and Protein Structures’, PLoS One, 9: e100400–8. 24936864 10.1371/journal.pone.0100400 PMC4061080

[veaa004-B23] Harris M. E. et al (2002) ‘Among 46 Near Full Length HIV Type 1 Genome Sequences from Rakai District, Uganda, Subtype D and AD Recombinants Predominate’, AIDS Research and Human Retroviruses, 18: 1281–90. 12487816 10.1089/088922202320886325

[veaa004-B24] Harris R. S. (2007) Improved Pairwise Alignment of Genomic DNA. University Park, PA: The Pennsylvania State University.

[veaa004-B25] Hartigan M., Wong J. A. (1979) ‘Algorithm as 136: A *K*-Means Clustering Algorithm’, Applied Statistics, 28: 100–8.

[veaa004-B26] Hu W. S., Hughes S. H. (2012) ‘HIV-1 Reverse Transcription’, Cold Spring Harbor Perspectives in Medicine, 2: 1–22. 10.1101/cshperspect.a006882 PMC3475395 23028129

[veaa004-B27] Hunt M. et al (2015) ‘IVA: Accurate De Novo Assembly of RNA Virus Genomes’, Bioinformatics, 31: 2374–6. 25725497 10.1093/bioinformatics/btv120 PMC4495290

[veaa004-B28] Joint United Nations Programme on HIV/AIDS. (2019) *UNAIDS DATA 2019*. Geneva: UNAIDS. doi: 10.1126/science.7716530. 12349391

[veaa004-B29] Kaleebu P. et al (2000) ‘Molecular Epidemiology of HIV Type 1 in a Rural Community in Southwest Uganda’, AIDS Research and Human Retroviruses, 16: 393–401. 10772525 10.1089/088922200309052

[veaa004-B30] Kalish M. L. et al (2004) ‘Recombinant Viruses and Early Global HIV-1 Epidemic’, Emerging Infectious Diseases, 10: 1227–34. 15324542 10.3201/eid1007.030904 PMC3323344

[veaa004-B31] Kalyaanamoorthy S. et al (2017) ‘ModelFinder: Fast Model Selection for Accurate Phylogenetic Estimates’, Nature Methods, 14: 587–9. 28481363 10.1038/nmeth.4285 PMC5453245

[veaa004-B32] Kassambara A., Mundt F. (2017) *factoextra: Extract and Visualize the Results of Multivariate Data Analyses* R Package Version 1.0.5, https://CRAN.R-project.org/package=factoextra.

[veaa004-B33] Katoh K., Standley D. M. (2013) ‘MAFFT Multiple Sequence Alignment Software Version 7: Improvements in Performance and Usability’, Molecular Biology and Evolution, 30: 772–80. 23329690 10.1093/molbev/mst010 PMC3603318

[veaa004-B34] Kiwanuka N. et al (2010) ‘HIV-1 Viral Subtype Differences in the Rate of CD4+ T-Cell Decline’, Journal of Acquired Immune Deficiency Syndromes (1999), 54: 180–4. 20010433 10.1097/QAI.0b013e3181c98fc0 PMC2877752

[veaa004-B35] Korber B. (2000) ‘Timing the Ancestor of the HIV-1 Pandemic Strains’, Science, 288: 1789–96. 10846155 10.1126/science.288.5472.1789

[veaa004-B36] Kosakovsky Pond S. L. et al (2009) ‘An Evolutionary Model-Based Algorithm for Accurate Phylogenetic Breakpoint Mapping and Subtype Prediction in HIV-1’, PLoS Computational Biology, 5: e1000581–21. 19956739 10.1371/journal.pcbi.1000581 PMC2776870

[veaa004-B37] Kosakovsky Pond S. L. et al (2018) ‘HIV-TRACE (Transmission Cluster Engine): A Tool for Large Scale Molecular Epidemiology of HIV-1 and Other Rapidly Evolving Pathogens’, Molecular Biology and Evolution, 35: 1812–9. 29401317 10.1093/molbev/msy016 PMC5995201

[veaa004-B38] Kraft C. S. et al (2012) ‘Timing and Source of Subtype-C HIV-1 Superinfection in the Newly Infected Partner of Zambian Couples with Disparate Viruses’, Retrovirology, 9: 22. 22433432 10.1186/1742-4690-9-22 PMC3349552

[veaa004-B39] Kuhanen J. (2010) ‘Sexualised Space, Sexual Networking & the Emergence of AIDS in Rakai, Uganda’, Health & Place, 16: 226–35. 19884035 10.1016/j.healthplace.2009.10.001

[veaa004-B40] Lee G. Q. et al (2017) ‘Prevalence and Clinical Impacts of HIV-1 Intersubtype Recombinants in Uganda Revealed by Near-Full-Genome Population and Deep Sequencing Approaches’, AIDS, 31: 2345–54. 28832407 10.1097/QAD.0000000000001619 PMC5656522

[veaa004-B41] Li H. et al (2009) ‘The Sequence Alignment/Map Format and SAMtools’, Bioinformatics, 25: 2078–9. 19505943 10.1093/bioinformatics/btp352 PMC2723002

[veaa004-B42] Li X. et al (2017) ‘Tracing the Epidemic History of HIV-1 CRF01-AE Clusters Using Near-Complete Genome Sequences’, Scientific Reports, 7: 1–11. 28642469 10.1038/s41598-017-03820-8 PMC5481428

[veaa004-B43] Lihana R. W. et al (2012) ‘Update on HIV-1 Diversity in Africa: A Decade in Review’, AIDS Reviews, 14: 83–100. 22627605

[veaa004-B44] Liljeros F. et al (2001) ‘The Web of Human Sexual Contacts’, Nature, 411: 907–8. 11418846 10.1038/35082140

[veaa004-B45] Maechler M. et al (2019) Cluster: Cluster Analysis Basics and Extensions. R Package Version 2.0.8, https://cran.r-project.org/package=cluster.

[veaa004-B46] Marozsan A. J. et al (2005) ‘Differences in the Fitness of Two Diverse Wild-Type Human Immunodeficiency Virus Type 1 Isolates Are Related to the Efficiency of Cell Binding and Entry’, Journal of Virology, 79: 7121–34. 15890952 10.1128/JVI.79.11.7121-7134.2005 PMC1112120

[veaa004-B47] McVean G., Awadalla P., Fearnhead P. (2002) ‘A Coalescent-Based Method for Detecting and Estimating Recombination From Gene Sequences’, Genetics, 160: 1231–41. 11901136 10.1093/genetics/160.3.1231 PMC1462015

[veaa004-B48] Mehta S. R. et al (2015) ‘HIV Transmission Networks in the San Diego-Tijuana Border Region’, EBioMedicine, 2: 1456–63. 26629540 10.1016/j.ebiom.2015.07.024 PMC4634195

[veaa004-B49] Ministry of Health Uganda. (2019) *Uganda Population-Based HIV Impact Assessment (UPHIA) 2016–2017: Final Report*. Kampala: Ministry of Health, Govt. of Uganda.

[veaa004-B50] Nguyen L. T. et al (2015) ‘IQ-TREE: A Fast and Effective Stochastic Algorithm for Estimating Maximum-Likelihood Phylogenies’, Molecular Biology and Evolution, 32: 268–74. 25371430 10.1093/molbev/msu300 PMC4271533

[veaa004-B51] Niama F. R. et al (2006) ‘HIV-1 Subtypes and Recombinants in the Republic of Congo’, Infection, Genetics and Evolution, 6: 337–43. 10.1016/j.meegid.2005.12.001 16473564

[veaa004-B52] Olabode A. S. et al (2019) ‘Evidence for a Recombinant Origin of HIV-1 Group M from Genomic Variation’, Virus Evolution, 5: 1–8. 10.1093/ve/vey039 PMC6342232 30687518

[veaa004-B53] Paradis E., Schliep K. (2019) ‘Ape 5.0: An Environment for Modern Phylogenetics and Evolutionary Analyses in R’, Bioinformatics, 35: 526–8. 30016406 10.1093/bioinformatics/bty633

[veaa004-B54] Pillay D. et al (2015) ‘PANGEA-HIV: Phylogenetics for Generalised Epidemics in Africa’, The Lancet Infectious Diseases, 15: 259–61. 25749217 10.1016/S1473-3099(15)70036-8 PMC4968650

[veaa004-B55] Pineda-Peña A. C. et al (2013) ‘Automated Subtyping of HIV-1 Genetic Sequences for Clinical and Surveillance Purposes: Performance Evaluation of the New REGA Version 3 and Seven Other Tools’, Infection, Genetics and Evolution, 19: 337–48. 10.1016/j.meegid.2013.04.032 23660484

[veaa004-B56] Rambaut A. et al (2001) ‘Phylogeny and the Origin of HIV-1’, Nature, 410: 1047–8. 11323659 10.1038/35074179

[veaa004-B57] Rambaut A. et al (2004) ‘The Causes and Consequences of HIV Evolution’, Nature Reviews Genetics, 5: 52–61. 10.1038/nrg1246 14708016

[veaa004-B58] R Core Team. (2019) R: A Language and Environment for Statistical Computing. Vienna, Austria: R Foundation for Statistical Computing.

[veaa004-B59] Redd A. D. et al (2012) ‘The Rates of HIV Superinfection and Primary HIV Incidence in a General Population in Rakai, Uganda’, The Journal of Infectious Diseases, 206: 267–74. 22675216 10.1093/infdis/jis325 PMC3415936

[veaa004-B60] Redd A. D. et al (2014) ‘The Rates of HIV-1 Superinfection and Primary HIV-1 Infection are Similar in Female Sex Workers in Uganda’, AIDS, 28: 2147–52. 25265078 10.1097/QAD.0000000000000365 PMC4921228

[veaa004-B61] Revell L. J. (2012) ‘Phytools: An R Package for Phylogenetic Comparative Biology (and Other Things)’, Methods in Ecology and Evolution, 3: 217–23.

[veaa004-B62] Robertson D. L. (2000) ‘HIV-1 Nomenclature Proposal HIV-1 Nomenclature Proposal HIV-1 Nomenclature Proposal’, Science, 288: 55. 10766634 10.1126/science.288.5463.55d

[veaa004-B63] Robertson D. L. et al (1995) ‘Recombination in HIV-1’, Nature, 374: 124–6. 10.1038/374124b0 7877682

[veaa004-B64] Salminen M. et al (1995) ‘Identification of Breakpoints in Intergenotypic Recombinants of HIV Type 1 by Bootscanning’, AIDS Research and Human Retroviruses, 11: 1423–5. 8573403 10.1089/aid.1995.11.1423

[veaa004-B65] Serwadda D. et al (1985) ‘Slim Disease: A New Disease in Uganda and Its Association with HTLV-III Infection’, The Lancet, 326: 849–52. 10.1016/S0140-6736(85)90122-9 2864575

[veaa004-B66] Serwadda D. et al (1986) ‘Further Experience with Kaposi’s Sarcoma in Uganda’, British Journal of Cancer, 53: 497–500. 3011052 10.1038/bjc.1986.78 PMC2001431

[veaa004-B67] Simmonds P. et al (1990) ‘Analysis of Sequence Diversity in Hypervariable Regions of the External Glycoprotein of Human Immunodeficiency Virus Type 1’, Journal of Virology, 64: 5840–50. 2243378 10.1128/jvi.64.12.5840-5850.1990 PMC248744

[veaa004-B68] Simon-Loriere E. et al (2009) ‘Molecular Mechanisms of Recombination Restriction in the Envelope Gene of the Human Immunodeficiency Virus’, PLoS Pathogens, 5: e1000418. 19424420 10.1371/journal.ppat.1000418 PMC2671596

[veaa004-B69] Simon-Loriere E. et al (2010) ‘RNA Structures Facilitate Recombination-Mediated Gene Swapping in HIV-1’, Journal of Virology, 84: 12675–82. 20881047 10.1128/JVI.01302-10 PMC3004330

[veaa004-B70] Smyth R. P., Davenport M. P., Mak J. (2012) ‘The Origin of Genetic Diversity in HIV-1’, Virus Research, 169: 415–29. 22728444 10.1016/j.virusres.2012.06.015

[veaa004-B71] Song H. et al (2018) ‘Tracking HIV-1 Recombination to Resolve Its Contribution to HIV-1 Evolution in Natural Infection’, Nature Communications, 9:1928. 10.1038/s41467-018-04217-5 PMC5954121 29765018

[veaa004-B72] Ssemwanga D. et al (2011) ‘Multiple HIV-1 Infections with Evidence of Recombination in Heterosexual Partnerships in a Low Risk Rural Clinical Cohort in Uganda’, Virology, 411: 113–31. 21239033 10.1016/j.virol.2010.12.025 PMC3041926

[veaa004-B73] Ssemwanga D. et al (2012) ‘HIV Type 1 Subtype Distribution, Multiple Infections, Sexual Networks, and Partnership Histories in Female Sex Workers in Kampala, Uganda’, AIDS Research and Human Retroviruses, 28: 357–65. 21749285 10.1089/aid.2011.0024

[veaa004-B74] Struck D. et al (2014) ‘COMET: Adaptive Context-Based Modeling for Ultrafast HIV-1 Subtype Identification’, Nucleic Acids Research, 42: 1–11. 25120265 10.1093/nar/gku739 PMC4191385

[veaa004-B75] Tamura K., Nei M. (1993) ‘Estimation of the Number of Nucleotide Substitutions in the Control Region of Mitochondrial DNA in Humans and Chimpanzees’, Molecular Biology and Evolution, 10: 512–26. 8336541 10.1093/oxfordjournals.molbev.a040023

[veaa004-B76] Taylor J. E., Korber B. T. (2005) ‘HIV-1 Intra-Subtype Superinfection Rates: Estimates Using a Structured Coalescent With Recombination’, Infection, Genetics and Evolution, 5: 85–95. 10.1016/j.meegid.2004.07.001 15567142

[veaa004-B77] Tibshirani R., Walther G., Hastie T. (2001) ‘Estimating the Number of Clusters in a Data Set via the Gap Statistic’, Journal of the Royal Statistical Society: Series B (Statistical Methodology), 63: 411–23.

[veaa004-B78] Turk G., Carobene M. G. (2015) ‘Deciphering How HIV-1 Intersubtype Recombination Shapes Viral Fitness and Disease Progression’, EBioMedicine, 2: 188–9. 26137559 10.1016/j.ebiom.2015.02.011 PMC4484815

[veaa004-B79] Ward M. J. et al (2013) ‘Estimating the Rate of Intersubtype Recombination in Early HIV-1 Group M Strains’, Journal of Virology, 87: 1967–73. 23236072 10.1128/JVI.02478-12 PMC3571495

[veaa004-B80] Woo J., Robertson D. L., Lovell S. C. (2014) ‘Constraints From Protein Structure and Intra-Molecular Coevolution Influence the Fitness of HIV-1 Recombinants’, Virology, 454–5: 34–9. 10.1016/j.virol.2014.01.029 24725929

[veaa004-B81] Worobey M. et al (2008) ‘Direct Evidence of Extensive Diversity of HIV-1 in Kinshasa by 1960’, Nature, 455: 661–4. 18833279 10.1038/nature07390 PMC3682493

[veaa004-B82] Yang C. et al (2004) ‘Genetic Diversity and High Proportion of Intersubtype Recombinants Among HIV Type 1-Infected Pregnant Women in Kisumu, Western Kenya’, AIDS Research and Human Retroviruses, 20: 565–74. 15186532 10.1089/088922204323087822

[veaa004-B83] Yang O. O. et al (2005) ‘Human Immunodeficiency Virus Type 1 Clade B Superinfection: Evidence for Differential Immune Containment of Distinct Clade B Strains’, Journal of Virology, 79: 860–8. 15613314 10.1128/JVI.79.2.860-868.2005 PMC538553

[veaa004-B84] Yebra G. et al (2015) ‘Analysis of the History and Spread of HIV-1 in Uganda Using Phylodynamics’, Journal of General Virology, 96: 1890–8. 25724670 10.1099/vir.0.000107 PMC4635457

[veaa004-B85] Yirrell D. L. et al (1998) ‘Molecular Epidemiological Analysis of HIV in Sexual Networks in Uganda’, AIDS, 12: 285–90. 9517991 10.1097/00002030-199803000-00006

[veaa004-B86] Yirrell D. L. et al (2002) ‘Inter- and Intra-Genic Intersubtype HIV-1 Recombination in Rural and Semi-Urban Uganda’, AIDS, 16: 279–86. 11807313 10.1097/00002030-200201250-00018

