# Supplementary material for: Pervasive and non-random recombination in near full-length HIV genomes from Uganda
Source: Virus Evol. 2020 Feb 11;6(1):veaa004. doi: 10.1093/ve/veaa004 (PMC7204518; doi:10.1093/ve/veaa004)
Supplement: veaa004_Supplementary_Data [file veaa004_supplementary_data.zip › Final_supplementary_info_10jan2020.docx]

**Supplementary information**

**Supplementary Table 1: subtyping results from COMET, REGA, and SCUEAL, and the agreement between them.**

[TableS1.csv]

**Figure S1: SCUEAL sensitivity**

Figure S1: For each of ten *in-silico* inter-subtype recombinants, and the number of inter-subtype breakpoints found for each 100bp-region along the genome in 100 replicate SCUEAL assessments.

*In silico* A1/D inter-subtype recombinants, with a random number of breakpoints from 1 to 3, random breakpoint locations, and a random selection of three “pure” A1 (labelled Ax, Ay, Az) and three “pure” D subtype sequences (labelled Dx, Dy, Dz) taken from the PANGEA dataset (pre-screened with SCUEAL) were created. Each *in silico* recombinant was analysed by SCUEAL 100 times.

The majority of the SCUEAL replicates found inter-subtype breakpoints as expected. Examples 3, 4, 5, and 10 worked particularly consistently (100/100 replicates found breakpoints in the same 100bp region as expected). More difficult scenarios, (examples 1 and 7 with small recombination fragments) still performed well finding breakpoints most of the time (60 or more/100), with other breakpoints close by.

**Supplementary Table 2: SCUEAL reference set used**

B_FR_1983_HXB2_LAI_IIIB_BRU_K03455 , A1_KE_2001_ML1901PCR2_EU110095 , A1_RU_2000_RU00051_EF545108 , A1_RW_1992_92RW025A_AB287377 , A1_AU_2004_PS1044_Day177_DQ676873 , A1_ZA_2004_04ZASK162B1_DQ396400 , A1_CD_1997_97CD_KTB13_AM000054 , A1_CD_1997_97CD_KCC2_AM000053 , A1_CD_2002_02CD_KTB035_AM000055 , A1_UG_1998_98UG57135_AF484508 , A1_SN_2001_DDJ369_AY521631 , A1_KE_2000_NKU3005_AF457089 , A1_KE_2000_MSA4070_AF457081 , A1_KE_2000_MSA4069_AF457080 , A1_KE_2000_KSM4030_AF457079 , A1_KE_2000_KNH1209_AF457069 , A1_KE_2000_KNH1207_AF457068 , A1_KE_2000_KER2012_AF457055 , A1_KE_2000_KER2008_AF457052 , A1_SE_1995_SE8891_AF069673 , A1_SE_1995_SE8538_AF069669 , A1_KE_1994_Q23_17_AF004885 , A2_CD_1997_97CDKTB48_AF286238 , A2_CY_1994_94CY017_41_AF286237 , A3_IT_2000_IT070_DQ445119 , B_GE_2003_03GEMZ010_DQ207942 , B_KR_2004_04LHS6_AY839827 , B_DK_2004_PMVL_013_EF514700 , B_BR_2003_BREPM1024_EF637056 , B_BR_2003_BREPM1033_EF637050 , B_BR_2003_BREPM1040_EF637047 , B_JP_2001_DR388_AB289590 , B_JP_2000_DR2510_AB287372 , B_JP_2005_DR7065_AB287368 , B_AU_2003_PS2008_Day182_DQ676875 , B_BR_2002_02BR011_DQ358809 , B_RU_2004_04RU139089_AY751407 , B_CA___GOL016V01_DQ322223 , B_JP_2004_04JPDR6075B_AB221126 , B_MM_1999_mSTD101_AB097870 , B_AR_2004_04AR151516_DQ383752 , B_AR_2000_ARMS008_AY037269 , B_CA_1998_CANC10FULL_AY779557 , B_EC_1989_EC102_AY173960 , BF_UY_2001_01UYTRA1020_AY781128 , BG_DE_2001_9196_01_AY882421 , C_ZA_2002_02ZAPS015MB1_DQ369995 , C_ZA_2003_03ZAPS052MB1_DQ369987 , C_ZA_2003_03ZAPS049MB1_DQ369986 , C_ZM_2002_02ZMBC_AB254149 , C_ZM_2002_02ZM108_AB254141 , C_ZA_2004_04ZAPS169MB1_DQ396393 , C_ZA_2003_03ZAPS125MB1_DQ396390 , C_ZA_2003_03ZAPS112MB2_DQ396386 , C_ZA_2003_03ZAPS131MB1_DQ396380 , C_ZA_2003_03ZAPS056MB1_DQ396374 , C_ZA_2003_03ZAPS105MB2_DQ445632 , C_ZA_2002_02ZAPS005MB1_DQ351235 , C_ZA_2003_03ZASK233B1_DQ351234 , C_ZA_2003_03ZAPS025MB1_DQ351226 , C_ZA_2002_02ZAPS006MB1_DQ351220 , C_ZA_2003_03ZAPS130MB1_DQ275658 , C_ZA_2003_03ZAPS073MB1_DQ275649 , C_ZA_2004_04ZAPS195B1_DQ164118 , C_ZA_2003_03ZASK213B1_DQ093607 , C_ZA_2004_04ZAPS190B1_DQ093602 , C_ZA_2003_03ZASK211B1_DQ093601 , C_ZA_2004_04ZASK170B1_DQ093595 , C_ZA_2003_03ZAPS079B1_DQ093592 , C_ZA_2003_03ZASK084B1_AY901981 , C_ZA_2003_03ZASK073B1_AY901970 , C_ZA_2003_03ZASK061B1_AY901968 , C_ZA_2003_03ZASK020B2_AY878064 , C_ZA_2000_1217MB_AY463226 , C_ZA_2000_1170MB_AY463225 , C_ZA_2000_1195MB_AY463220 , C_ZA_2000_1134MB_AY463217 , C_KE_1991_KNH1268_AY945738 , C_TZ_2002_CO6130_AY734559 , C_TZ_2002_CO3305_AY734558 , C_TZ_2002_CO328_AY734551 , C_YE_2002_02YE511_AY795906 , C_AR_2001_ARG4006_AY563170 , C_ZA_2000_1184MB_AY838566 , C_TZ_2001_BD22_11_AY253321 , C_TZ_2001_A388_AY253317 , C_TZ_2001_A301_AY253312 , C_TZ_2001_A207_AY253307 , C_IL_1999_99ET14_AY255825 , C_IL_1999_99ET1_AY255823 , C_ZA_2001_01ZATM45_AY228557 , C_BW_2000_00BW38428_AF443109 , C_BW_2000_00BW2127214_AF443105 , C_BW_2000_00BW20872_AF443104 , C_BW_2000_00BW20636_AF443103 , C_BW_2000_00BW192113_AF443101 , C_BW_2000_00BW17956_AF443097 , C_BW_2000_00BW17835_AF443096 , C_BW_2000_00BW17593_AF443094 , C_BW_2000_00BW1686_AF443093 , C_BW_2000_00BW16162_AF443092 , C_BW_2000_00BW087421_AF443090 , C_BW_2000_00BW076820_AF443089 , C_BW_2000_00BW07621_AF443088 , C_BW_1999_99BWMC168_AF443087 , C_BW_1998_98BWMO18D5_AF443080 , C_ZA_1998_CTSC2_AY043176 , C_TZ_1997_97TZ04_AF361874 , C_BW_1996_96BW0502_AF110967 , C_BW_1996_96BW0402_AF110962 , C_ZM_1996_96ZM651_AF286224 , C_IN_1994_94IN11246_AF067159 , C_ET_1986_ETH2220_U46016 , D_CM_2001_01CM_0009BBY_AY371155 , D_YE_2002_02YE516_AY795907 , D_YE_2001_01YE386_AY795903 , D_KR_2004_04KBH8_DQ054367 , D_ZA_1986_R482_AY773341 , D_ZA_1985_R286_AY773340 , D_ZA_1985_R214_AY773339 , D_UG_1998_98UG57131_AF484505 , D_UG_1998_98UG57130_AF484504 , D_UG_1999_99UGF05734_AF484490 , D_TZ_2001_A280_AY253311 , D_KE_2001_NKU3006_AF457090 , D_CD_1983_ELI_K03454 , F1_ES___P1146_DQ979023 , F1_AR_2002_ARE933_DQ189088 , F1_FR_1996_96FR_MP411_AJ249238 , F1_BE_1993_VI850_AF077336 , F2_CM_1995_95CM_MP255_AJ249236 , G_CU_1999_Cu74_AY586547 , G_PT___PT2695_AY612637 , G_CM_2001_01CM_4049HAN_AY371121 , G_NG_2001_PL0567_DQ168573 , G_IT_2000_IT031_DQ445117 , G_CM_1996_96CMABB55_AY772535 , G_BE_1996_DRCBL_AF084936 , G_KE_1993_HH8793_1_1_AF061640 , H_BE_1993_VI991_AF190127 , H_CF_1990_056_AF005496 , J_CD_1997_J_97DC_KTB147_EF614151 , J_SE_1994_SE9173_7022_AF082395 , K_CM_1996_96CM_MP535_AJ249239 , 01_AE_CN_2005_Fj065_EF036534 , 01_AE_CN_2005_Fj057_EF036530 , 01_AE_CN_2005_Fj052_EF036528 , 01_AE_CN_2006_FJ054_DQ859180 , 01_AE_CF_1990_90CF4071_AF197341 , 02_AG_CM_1999_pBD6_15_AY271690 , 02_AG_NG___IBNG_L39106 , 03_AB_RU_1997_KAL153_2_AF193276 , 03_AB_BY_2000_98BY10443_AF414006 , 04_cpx_CY_1994_94CY032_3_AF049337 , 04_cpx_GR_1997_GR84_97PVMY_AF119819 , 05_DF_ES_1999_X492_AY227107 , 05_DF_BE___VI1310_AF193253 , 06_cpx_GH_2003_03GH173_06_AB286851 , 06_cpx_EE_2001_EE0359_AY535659 , 06_cpx_AU_1996_BFP90_AF064699 , 07_BC_CN_2005_XJDC6431_2_EF368372 , 07_BC_CN_2005_XJDC6441_EF368370 , 08_BC_CN_1997_97CNGX_6F_AY008715 , 09_cpx_GH_1996_96GH2911_AY093605 , 09_cpx_CI_2000_00IC_10092_AJ866553 , 10_CD_TZ_1996_96TZ_BF071_AF289549 , 10_CD_TZ_1996_96TZ_BF061_AF289548 , 11_cpx_CM_1997_MP818_AJ291718 , 11_cpx_CM_1995_95CM_1816_AF492624 , 11_cpx_CM_1996_96CM_4496_AF492623 , 12_BF_AR_1997_A32879_AF408629 , 12_BF_AR_1999_ARMA159_AF385936 , 13_cpx_CM_2002_02CM_A1394_DQ845388 , 13_cpx_CM_2004_04CM_632_28_DQ845387 , 13_cpx_CM_1996_96CM_1849_AF460972 , 14_BG_ES_2000_X623_AF450097 , 14_BG_ES_2000_X605_AF450096 , 15_01B_TH_1996_M169_DQ354120 , 15_01B_TH_1999_99TH_MU2079_AF516184 , 15_01B_TH_1999_99TH_R2399_AF530576 , 16_A2D_KE_1991_KNH1271_AY945736 , 16_A2D_KR_1997_97KR004_AF286239 , 17_BF_PE_2002_PE02_PCR0155_EU581828 , 17_BF_AR_2002_AR02_ARG1139_EU581825 , 18_cpx_CU_1999_CU14_AY586541 , 18_cpx_CM_1997_CM53379_AF377959 , 19_cpx_CU_1999_CU29_AY588971 , 20_BG_CU_1999_Cu103_AY586545 , 20_BG_ES_1999_R77_AY586544 , 21_A2D_KE_1991_KNH1254_AY945737 , 22_01A1_CM_2001_01CM_0001BBY_AY371159 , 23_BG_CU_2003_CB347_AY900572 , 23_BG_CU_2003_CB118_AY900571 , 24_BG_CU_2003_CB378_AY900574 , 25_cpx_SA_2003_J11233_EU697906 , 25_cpx_CM_2006_06CM_BA_040_EU693240 , 27_cpx_FR_2004_04CD_FR_KZS_AM851091 , 27_cpx_CD_1997_97CDKTB49_AJ404325 , 28_BF_BR_1999_BREPM12609_DQ085873 , 28_BF_BR_1999_BREPM12313_DQ085872 , 29_BF_BR_2001_BREPM16704_DQ085876 , 31_BC_BR_2002_110PA_EF091932 , 31_BC_BR_2004_04BR142_AY727527 , 32_06A1_EE_2001_EE0369_AY535660 , 33_01B_MY_2005_05MYKL007_1_DQ366659 , 34_01B_TH_1999_OUR2478P_EF165541 , 35_AD_AF_2005_05AF026_EF158043 , 36_cpx_CM_2000_00CMNYU830_EF087994 , 37_cpx_CM_1997_CM53392_AF377957 , 39_BF_BR_2003_03BRRJ103_EU735534 , 40_BF_BR_2005_05BRRJ200_EU735539 , 40_BF_BR_2004_04BRRJ115_EU735538 , 43_02G_SA_2003_J11223_EU697904 , 47_BF_ES_2008_P1942_GQ372987 , N_CM_2004_04CM_1015_04_DQ017382 , N_CM_1995_YBF30_AJ006022 , O_CM_1999_99CMU4122_AY169815 , O_BE_1987_ANT70_L20587

**Supplementary Figure S2: Pilot test for identification of homologous breakpoints**

To test the sliding window pairwise linkage approach described in the methods (section 2.4), dummy recombinants were generated using the SCUEAL reference genomes to show that recombinants with the sections of the same subtype would be linked. Twenty of these recombinants were generated with a random selection of genomes (23 A1 SCUEAL reference genomes and 12 D SCUEAL reference genomes labelled A1:A23, D24:D35) and a random number of breakpoints between 1 and 3. Fig1 shows these recombinants in the pairwise window linkage analysis, where windows containing the same subtype were successfully linked. Note that at the 2% level, some windows will match even with different reference subtypes, (e.g. references A4 and A20 matched in window 13 in pair 5), but this was linkage in a single window only.


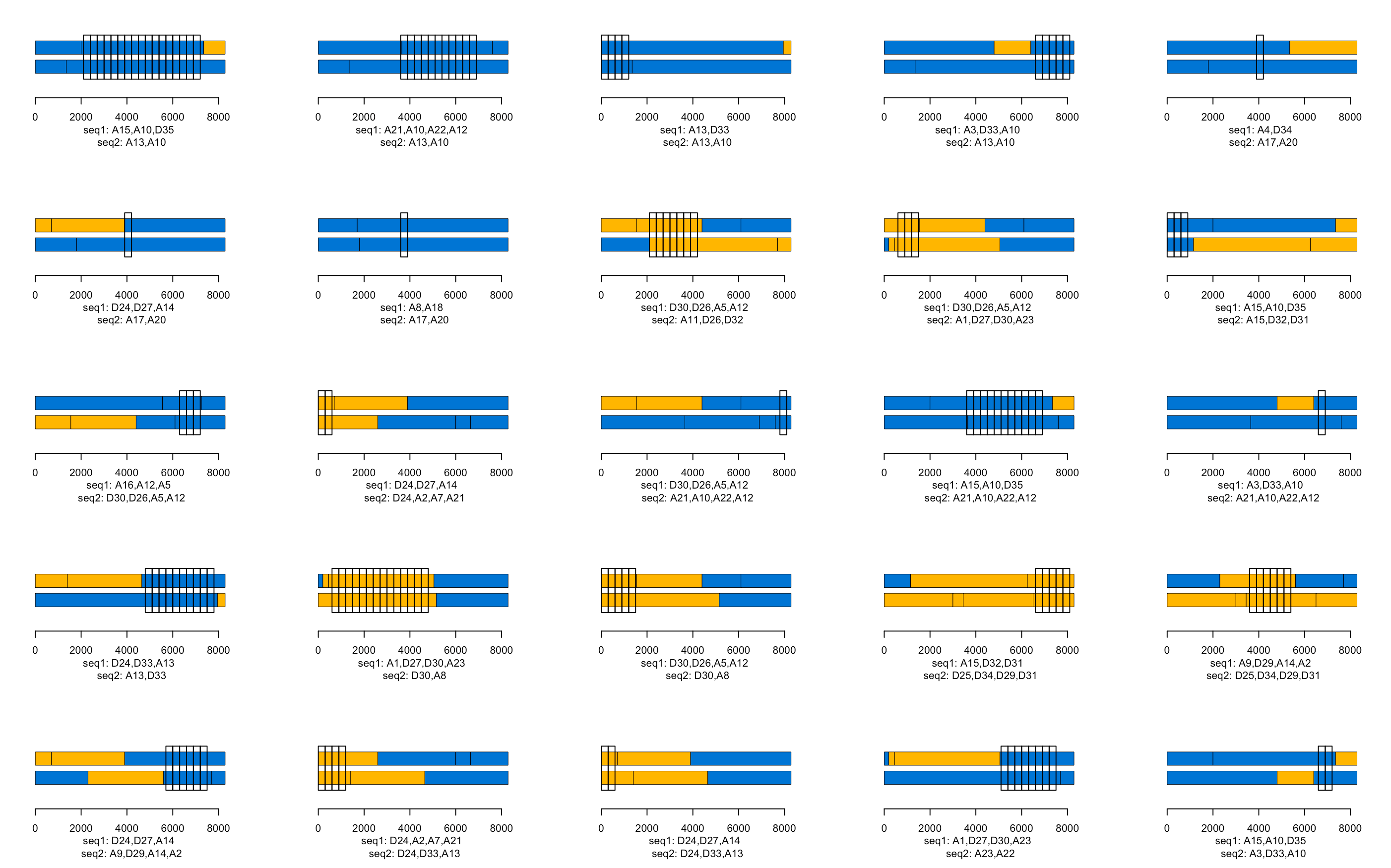


Fig S2: Pairs of randomly generated recombinant genomes linked by a distance of less than 2% (TN93) in more than one window along the genomes. The matching windows are shown with open clear boxes, and the SCUEAL subtyping result is in colour (blue for Subtype A1 and orange for Subtype D).

**Supplementary Figure S3:** Maximum-likelihood reconstruction of the non-A1/D recombinants using IQ-TREE (n=68), midpoint rooted, and their SCUEAL subtype assessment (right). A phylogenetic tree of recombinants clearly violates the assumption of absence of recombination, but is used to show the absence of any clear CRF.

**Supplementary information: *K*-means clustering justification**

Fig.S4 shows three methods for determining the optimal number of clusters. The gap statistic (top left) compares the within cluster variation for each value of k against a null reference distribution. Here 9 appears to be most appropriate value of k. The elbow method (top right) plots all within-cluster sum of squares for each value of k, and the bend or elbow in the plot is an indicator of the appropriate number of clusters. There is a curve between k=2 and 5 without an obvious elbow, however there is a kink at k=9. The silhouette method (bottom left) shows the average silhouette width (a measure of how tightly grouped genomes within each cluster are), and k=2, k=3, k=5 or k=9 have higher silhouette width.

Figure S4: Cluster finding metrics, top to bottom, left to right, a) Gap statistic b) Elbow method c) Silhouette method and d) visualisation of the raw Euclidean distance matrix.

Fig.S5 shows genomes clustered with k=2, k=3, k=5 and k=9. Using k=2 broadly splits the genomes into predominantly subtype A1, and subtype D genomes, k=3 splits the genome into mostly A1, mostly D, and D genomes with an A1 envelope region. Using k=5 adds another group of A1 genomes with a D envelope region, and using k=9 adds a few smaller groups with clustering of subtype D in gag and pol regions. The broad scale pattern of interest (that is the intact inheritance of partial envelope) is evident in k=3, k=5 and k=9.

FigS5: Groupings of the A1/D recombinants clustered by kmeans clustering with alternative values of k (2, 3, 5, 9). Segments of orange colour represent Subtype D, while blue colouration represents subtype A1.

FigS6. Four alternative k=9 groupings. The *K*-means clustering algorithm is stochastic in that it takes a random selection of k genomes as starting point. There are overall similar patterns albeit with subtle differences.
